# Learned parametrized dynamic movement primitives with shared synergies for controlling robotic and musculoskeletal systems

**DOI:** 10.3389/fncom.2013.00138

**Published:** 2013-10-17

**Authors:** Elmar Rückert, Andrea d'Avella

**Affiliations:** ^1^Institute for Theoretical Computer Science, Graz University of TechnologyAustria; ^2^Laboratory of Neuromotor Physiology, Fondazione Santa LuciaRome, Italy

**Keywords:** dynamic movement primitives, muscle synergies, reinforcement learning, motor control, musculoskeletal model

## Abstract

A salient feature of human motor skill learning is the ability to exploit similarities across related tasks. In biological motor control, it has been hypothesized that muscle synergies, coherent activations of groups of muscles, allow for exploiting shared knowledge. Recent studies have shown that a rich set of complex motor skills can be generated by a combination of a small number of muscle synergies. In robotics, dynamic movement primitives are commonly used for motor skill learning. This machine learning approach implements a stable attractor system that facilitates learning and it can be used in high-dimensional continuous spaces. However, it does not allow for reusing shared knowledge, i.e., for each task an individual set of parameters has to be learned. We propose a novel movement primitive representation that employs parametrized basis functions, which combines the benefits of muscle synergies and dynamic movement primitives. For each task a superposition of synergies modulates a stable attractor system. This approach leads to a compact representation of multiple motor skills and at the same time enables efficient learning in high-dimensional continuous systems. The movement representation supports discrete and rhythmic movements and in particular includes the dynamic movement primitive approach as a special case. We demonstrate the feasibility of the movement representation in three multi-task learning simulated scenarios. First, the characteristics of the proposed representation are illustrated in a point-mass task. Second, in complex humanoid walking experiments, multiple walking patterns with different step heights are learned robustly and efficiently. Finally, in a multi-directional reaching task simulated with a musculoskeletal model of the human arm, we show how the proposed movement primitives can be used to learn appropriate muscle excitation patterns and to generalize effectively to new reaching skills.

## 1. Introduction

Reinforcement Learning of motor skills in robotics is considered to be very challenging due to the high-dimensional continuous state and action spaces. In many studies it has been shown that learning can be facilitated by the use of movement primitives (Schaal et al., 2003; Rückert et al., 2013). Movement primitives are parametrized representations of elementary movements, where typically for each motor skill a small set of parameters is tuned or learned. However, many motor control tasks are related and could be learned more effectively by exploiting shared knowledge.

This is a well-known concept in motor neuroscience, where muscle synergies or coherent activations of groups of muscles (d'Avella et al., 2003; d'Avella and Bizzi, 2005; Bizzi et al., 2008) have been proposed to simplify the control problem of complex musculoskeletal systems. In analyzing muscle activation recordings it has been demonstrated that by combining only few muscle activation patterns multiple task instances of natural motor behaviors, e.g., fast reaching movements of humans (d'Avella et al., 2006), primate grasping movements (Overduin et al., 2008), or walking patterns of infants, toddlers, and adults (Dominici et al., 2011) could be efficiently modeled. One important finding of theses studies is that the dimensionality of the motor control problem can be drastically reduced by reusing common knowledge of related tasks, i.e., grasping objects at different locations using a linear combination of shared muscle synergies. While this has been demonstrated in biological data analysis, only few robotic applications exist that use this shared task knowledge (Chhabra and Jacobs, 2006; Alessandro et al., 2012). These methods demonstrate the advantages of shared synergies in learning robotic tasks. However, different procedures were applied to obtain a parametric description of synergies, i.e., in Chhabra and Jacobs (2006) a variant of non-negative matrix factorization (d'Avella et al., 2003) was used given a set of pre-computed trajectories and in Alessandro et al. (2012) the synergies were extracted from dynamic responses of a robot system with random initialization. In contrast, we propose to learn the synergies representation in a reinforcement learning framework, where task-specific and task-invariant parameters in a multi-task learning setting are learned simultaneously.

In robotics the most widely used approach for motor skill learning are Dynamic Movement Primitives (DMPs) (Schaal et al., 2003; Ijspeert et al., 2013). This approach uses parametrized dynamical systems to determine a movement trajectory and has several benefits. First, as it is a model-free approach, there is no need to learn the typically non-linear, high-dimensional dynamic forward model of a robot (However, this is not the case when inverse dynamics controller are used to compute the control commands). Second, it provides a linear policy parametrization which can be used for imitation learning and policy search (Kober and Peters, 2011). The complexity of the trajectory can be scaled by the number of parameters (Schaal et al., 2003) and one can adapt meta-parameters of the movement such as the movement speed or the goal state (Pastor et al., 2009; Kober et al., 2010). Finally, the dynamical system is constructed such that the system is stable. This simplifies learning since even without modulating the dynamical system the movement trajectory is always attracted by a known (or learned) goal state. However, this parametrization does not allow for reusing shared knowledge, as proposed by the experimental findings studying complex musculoskeletal systems (d'Avella et al., 2003; Bizzi et al., 2008; d'Avella and Pai, 2010). Thus, typically for each motor task an individual movement parametrization has to be learned.

In this paper we propose to use a superposition of learned basis functions or synergies to modulate the stable attractor system of DMPs. This allows for reusing shared knowledge for learning multiple related tasks simultaneously while preserving the benefits of the dynamical systems, i.e., the stability in learning complex motor behavior. The synergies and their activation in time are learned from scratch in a standard reinforcement learning setup. Note that imitation learning could also be applied to implement an initial guess for the synergies, e.g., by using decomposition strategies discussed in d'Avella and Tresch (2001). However, this is beyond the scope of this paper. Moreover, our approach is like the DMPs applicable to discrete and rhythmic movements and allows for modeling time-varying synergies (d'Avella et al., 2006). We therefore denote our approach *DMPSynergies*. By using for each task a combination of individual, temporally fixed, basis functions DMPs can be modeled as special case of this approach. The benefit of the common prior knowledge is even more drastic when generalizing to new motor tasks given the previously learned basis functions. Thus, for simpler synergies only the weights for the linear combination have to be acquired and for time-varying synergies additionally the time-shift parameters need to be learned. This is demonstrated on a complex walking task and on reaching task using an arm actuated by muscles.

As in previous studies on DMPs (Meier et al., 2011; Mülling et al., 2013) we want to go beyond basic motor skills learning. However, in contrast to those studies that use a library of primitives for sequencing elementary movements (Meier et al., 2011) or mixing basic skills (Mülling et al., 2013), we implement the common shared knowledge among multiple tasks as prior in a hierarchical structure. On the lower level task related parameters, i.e., amplitude scaling weights or time-shift parameters are used to modulate a linear superposition of learned basis functions, the shared higher level knowledge. This has the promising feature that by combining just a small number of synergies diverse motor skills can be generated.

In the Materials and Methods, we will first briefly introduce DMPs (Schaal et al., 2003; Ijspeert et al., 2013) as we build on this approach. We then extend DMPs to allow for reusing shared task knowledge in the form of parametrized synergies. The advantage of the shared knowledge is evaluated in the Results on three multi-task learning scenarios. First, a simple via-point task is used to demonstrate the characteristics of the proposed representation. Then, rhythmic movements are learned in a dynamic 5-link planar biped walker environment. Finally, a musculoskeletal model of a human arm is used to evaluate our primitives on a muscle actuated system learning discrete reaching movements to multiple targets.

## 2. Materials and methods

### 2.1. Dynamic movement primitives

DMPs generate multi-dimensional trajectories by the use of non-linear differential equations (simple damped spring models) (Schaal et al., 2003). The basic idea is to use for each degree-of-freedom (DoF), or more precisely for each actuator, a globally stable, linear dynamical system of the form

(1) $$\tau \dot{z}=\alpha_{z}(\beta_{z}(g-y^{*})-z)+f,\;\tau {\dot{y}}^{*}=z,$$

which is modulated by a learnable non-linear function *f*. The final position of a movement is denoted by *g* and the variables y^*^ and ${\dot{y}}^{*}$ represent the desired state in i.e., joint angles and joint velocities. The time constants α and β are usually pre-defined. The temporal scaling factor τ can be used for de- or accelerating the movement execution as needed. Finally *z* denotes an internal variable of the dynamical system. For each DoF an individual function *f* is used which is different for discrete and rhythmic movements.

For discrete movements the function *f* only depends on the phase *s*, which is an abstraction of time and was introduced to scale the movement duration (Schaal et al., 2003). The function *f*(*s*) is constructed of the weighted sum of *N* Gaussian basis functions Ψ_*n*_

(2) $$f(s)=\frac{\sum_{n=1}^{N}\Psi_{n}(s)w_{n}s}{\sum_{n^{\prime }=1}^{N}\Psi_{n^{\prime }}(s)},$$

where for discrete movements these Gaussian basis functions are

$$\Psi_{n}(s)=\exp\left(-\frac{1}{2h_{n}^{2}}{\left(s-\mu_{n}\right)}^{2}\right),\;\tau \dot{s}=-\alpha_{s}s.$$

Only the weights *w*_*n*_ are parameters of the primitive which can modulate the shape of the movement. The centers or means μ_*n*_ ∈ [0, 1] specify at which phase of the movement the basis function becomes active. They are typically equally spaced in the range of *s* and not modified during learning. The bandwidth of the basis functions is given by *h*^2^_*n*_ and is typically chosen such that the Gaussians overlap.

For rhythmic movements periodic activation functions are used (Ijspeert et al., 2002). The non-linear function *f* reads

(3) $$f(\phi )=\frac{\sum_{n=1}^{N}\Psi_{n}(\phi )w_{n}}{\sum_{n^{\prime }=1}^{N}\Psi_{n^{\prime }}(\phi )},$$

where the periodic phase angle is denoted by ϕ ∈ [0, 2 π]. In Ijspeert et al. (2002) additionally a scalar variable was used to scale the amplitude of the oscillator, which was omitted for simplicity. The basis functions are given by

$$\Psi_{n}(\phi )=\exp(h_{n}(\text{cos}(\phi -\mu_{n})-1)),\;\tau \dot{\phi }=1,$$

which implement von Mises basis functions. Note that for the periodic basis functions the trajectory in Equation 1 oscillates around the attractor point or goal state *g*.

Integrating the dynamical systems in Equation 1 for each DoF results into a desired trajectory $\langle y_{t}^{*},{\dot{y}}_{t}^{*}\rangle$ of the joint angles. To follow this trajectory, in the most simple case a linear feedback controller is subsequently used to generate appropriate control commands denoted by **u**_*t*_:

(4) $$u_{t}=\text{diag}(k_{\text{pos}})(y_{t}^{*}-y_{t})+\text{diag}(k_{\text{vel}})({\dot{y}}_{t}^{*}-{\dot{y}}_{t}).$$

For each actuator the linear weights **W** = [**w**_1_, …, **w**_*D*_] as well as the control gains **k**_pos_ and **k**_vel_ have to be specified, i.e., θ = [**W**, **k**_pos_, **k**_vel_]. This results into *ND* + 2*D* parameters for the movement representation, where *D* denotes the number of actuators or muscles of a system. The simulated trajectory is denoted by $\langle y_{t},{\dot{y}}_{t}\rangle$.

In multi-task learning we want to learn *k* = 1..*K* tasks simultaneously. For very simply tasks, such as the via-point experiments described below, it could be sufficient to adapt the goal state *g*. However, this is usually not the case for more complex motor skill learning tasks in robotics. With DMPs typically for each motor skill an individual movement parametrization θ_*k*_ has to be learned. However, if we assume similarities among these tasks the learning problem could potentially be simplified by reusing shared knowledge. Inspired by experimental findings in biology (d'Avella et al., 2003; Bizzi et al., 2008; d'Avella and Pai, 2010) we extend these DMPs. Only the parametrization for the non-linear function *f*(*s*) for discrete movements or *f*(ϕ) for rhythmic movement changes. The dynamical system in Equation 1 and the linear feedback controller in Equation 4 remains the same.

### 2.2. Dynamic movement primitives with shared synergies (DMPSynergies)

For learning the *k*th task, we propose to use a linear combination of temporal flexible basis functions or synergies to parametrize the non-linear function *f*(*s*) in Equation 2 or for rhythmic movements *f*(ϕ) in Equation 3:

(5) $$f(s,k)=\sum_{m=1}^{M}\beta_{m,k}\Lambda \left(s,\theta_{m},\Delta s_{m,\;k}\right)s,$$

(6) $$f(\phi ,k)=\sum_{m=1}^{M}\beta_{m,k}\Omega \left(\phi ,\theta_{m},\Delta s_{m,\;k}\right),$$

where *s* denotes the phase variable which is only used for discrete movements. As with DMPs (Ψ_*n*_ in Equation 2) the functions Λ(.) and Ω(.) are different for discrete and rhythmic movements.

**Figure 1:**
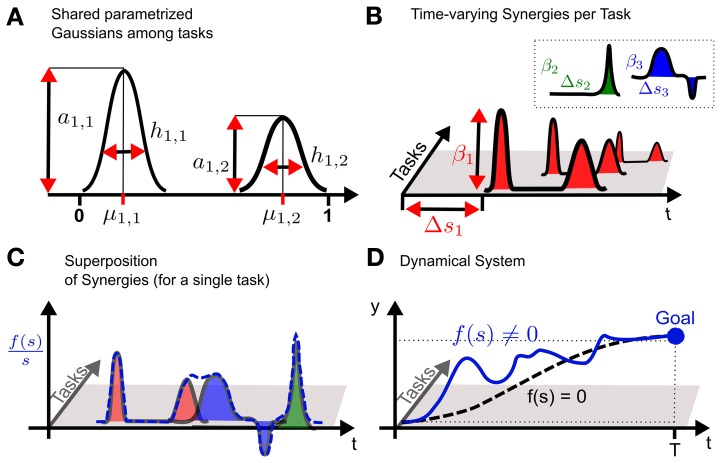
**Conceptual idea of using shared synergies in dynamical systems. (A)** A synergy is constructed by a superposition of parametrized Gaussians. The parameters are the amplitude *a*_*m*, *n*_, the mean μ_*m*, *n*_ and the bandwidth *h*_*m*, *n*_. In the example two Gaussians (*n* = 1..2) are used to model the first *m* = 1 synergy. **(B)** For each task only the activation β_*m*_ of a synergy is learned. Time-varying synergies additionally implement a time-shift Δ*s*_*m*_. The key concept is that multiple tasks share the same parametrized synergies shown in **(A)**, which represent task related common knowledge. **(C)** For each task the non-linear function *f*(*s*) is given by the weighted sum of the (time-shifted) synergies. Shown is a normalized version of *f*(*s*) to illustrate the effects of the superposition also at the end of the movement, which would usually converge toward zero. **(D)** Finally, the non-linear function *f*(*s*) is used to modulate a dynamical system. The unperturbed system with *f*(*s*) = 0 is denoted by the dashed line which is *attracted* by the goal state that is indicated by the large dot.

All *K* tasks share *m* = 1..*M* synergies which are parametrized via the vector θ_*m*_. Solely the weights β_*m*, *k*_ and the time-shift Δ*s*_*m*, *k*_ are individual parameters for each task. The basic concept of the model is sketched in Figure 1 for a one-dimensional discrete movement.

The complexity of each synergy is controlled by the number of Gaussians for discrete movements or by the number of von Mises basis functions for rhythmic patterns. We denote this number by *N*, where we parametrize in both cases the amplitude, the mean and the bandwidth. Thus, each synergy is represented by a parameter vector θ_*m*_ = [*a*_*m*, 1_, μ_*m*, 1_, *h*_*m*, 1_, …, *a*_*m*, *N*_, μ_*m*, *N*_, *h*_*m*, *N*_].

For discrete movements the function Λ(.) reads

(7) $$\Lambda \left(s,\theta_{m},\Delta s_{m,k}\right)=\sum_{n=1}^{N}a_{m,n}\text{exp}\left(-\frac{1}{2h_{m,n}{}^{2}}{\left(s-\mu_{m,n}+\Delta s_{m,k}\right)}^{2}\right).$$

For rhythmic movements a superposition of von Mises basis functions is used

(8) $$\Omega \left(\phi ,\theta_{m},\Delta s_{m,k}\right)=\sum_{n=1}^{N}a_{m,n}\text{exp}\left(h_{m,n}\text{cos}\left(\phi -\mu_{m,n}+\Delta s_{m,k}\right)-1\right).$$

DMPs (Schaal et al., 2003) can be modeled as a special case of this formulation. For DMPs using *n* =1.. *N* basis functions the mean μ_*m*, *n*_ and the bandwidth *h*_*m*, *n*_ of the basis functions are fixed as discussed in Section 2.1. Solely the *n* = 1..*N* amplitudes or weights *a*_*m*, *n*_ are learned. By fixing these parameters and by modeling the non-linear function *f*(*s*) for discrete movements or *f*(ϕ) for rhythmic movements using a single (*M* = 1) synergy our representation can be used to implement DMPs.

#### 2.2.1. Multi-dimensional systems

For multi-dimensional systems for each actuator *d* = 1..*D* an individual dynamical system in Equation 1 and hence an individual function *f*(*s*, *k*) in Equation 5 or *f*(ϕ, *k*) in Equation 6 is used (Schaal et al., 2003). The phase variable *s* or ϕ is shared among all DoF (Note that *k* = 1..*K* denotes the task.).

Extending our notation for multi-dimensional systems the non-linear function *f*(*s*, *k*) in Equation 5 can be written as

$$\underset{1\times 1}{\underset{︸}{f(s,d,k)}}=\sum_{m=1}^{M}\underset{1\times 1}{\underset{︸}{\beta_{m,k,\;d}}}\underset{1\times 1}{\underset{︸}{\Lambda \left(s,\theta_{m,\;d},\Delta s_{m,k,d}\right)s}}.$$

Depending on the dimension *d* different weights β_*m*, *k*, *d*_, policy vectors θ_*m*, *d*_ and time-shift parameters Δ*s*_*m*, *k*, *d*_ are used. Note that the policy vector θ_*m*, *d*_ is task-independent. Interestingly, when implementing additionally dimension-independent policy vectors, i.e., θ_*m*_ anechoic mixing coefficients (Giese et al., 2009) can be modeled.

Here, we only discuss discrete movement representations, however, the reformulation procedure applies also for rhythmic movement parametrizations. Let us also define a vector notation of **f**(*s*, *k*)

(9) $$\underset{1\times D}{\underset{︸}{f(s,k)}}=\sum_{m=1}^{M}\underset{1\times D}{\underset{︸}{\beta_{m,\;k}}}{}^{\circ}\underset{1\times D}{\underset{︸}{w_{m}\left(s,\theta_{m,1..D},\Delta s_{m,k,\;d}\right)}},$$

where the symbol ◦ denotes the Hadamard product, the element-wise multiplication of vectors. The synergy vectors are specified by

$$\begin{array}{l} w_{m}\left(s,\theta_{m,1..D},\Delta s_{m,\;k,d}\right)=[\Lambda \left(s,\theta_{m,1},\Delta s_{m,k,1}\right)s,\\ \;\Lambda \left(s,\theta_{m,2},\Delta s_{m,\;k,2}\right)s,\ldots ,\\ \;\Lambda \left(s,\theta_{m,D},\Delta s_{m,\;k,D}\right)s]. \end{array}$$

This vector notation is used in the following to compare to existing synergies representations (d'Avella et al., 2003, 2006).

### 2.3. Musculoskeletal models and muscle synergies

**Figure 2:**
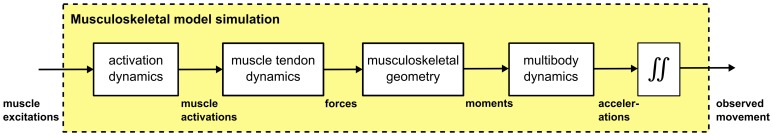
**Forward simulation of musculoskeletal models.** Muscle excitation patterns are used as input, which result in a delayed muscle activity response (activation dynamics). Muscle forces are the result of simulated muscle tendon dynamics, which are typically approximated by a Hill-type contractile element in series with tendon. These muscle forces are used to compute moments of force considering the musculoskeletal geometry. A physics engine is finally used to simulate multibody dynamics which are numerically integrated to generate movement trajectories.

We also use the proposed movement representation, the DMPSynergies, to generate muscle excitation patters. These patterns are applied as input in a forward simulation of a musculoskeletal model. A schematic overview of such a simulation is shown in Figure 2. We briefly discuss all processes involved.

**Muscle synergies for generating muscle excitation patterns** are used as input in forward dynamics simulations. In our simulation experiments we evaluate time-varying synergies (d'Avella et al., 2006), which are a particular instance of the DMPSynergies, i.e., the weights β_*m*, *k*_ and time-shift parameters Δ*s*_*m*, *k*_ in Equation 9 are independent of the dimension *d*. Thus, for discrete movements in multi-dimensional systems *f*(*s*, *k*) reads

(10) $$\underset{1\times D}{\underset{︸}{f(s,k)}}=\sum_{m=1}^{M}\underset{1\times 1}{\underset{︸}{\beta_{m,k}}}\underset{1\times D}{\underset{︸}{w_{m}\left(s+\Delta s_{m,\;k},\theta_{m,1..D},0\right)}},$$

where β_*m*, *k*_ is a scalar and the time-shift parameter Δ*s*_*m*, *k*_ is directly added to the phase variable *s*. This allows for a comparison to e.g., the formulation of time-varying synergies given in d'Avella et al. (2006), where

$$x(t,k)=\sum_{m=1}^{M}a_{m}^{k}v_{m}\left(t-t_{m}^{k}\right).$$

Shared synergies are represented by time-dependent vectors **v**_*m*_(*t* − *t*^*k*^_*m*_), where in contrast to the proposed DMPSynergies a minor difference is the sign of the time-shift parameter *t*^*k*^_*m*_.

In this formulation of time-varying synergies (d'Avella et al., 2006) only the time-invariant combination coefficients *a*^*k*^_*m*_ are task-dependent, whereas the vector **v**_*m*_ is task-independent. However, by using task, spatial or temporal (in)variant implementations of the mixing coefficients *a* or the basis vectors **v** other representations of synergies (d'Avella et al., 2003; Ivanenko et al., 2004; Giese et al., 2009) can be also implemented.

**Activation dynamics** model the effect of the delayed force generating process in muscles, as they are not capable of generating force instantaneously. Typically, for each muscle a first order differential equation is used, i.e., $\dot{a}=(f{\left(s,k\right)}^{2}-f(s,k)a)/\tau_{\text{rise}}+(f(s,k)-a)/\tau_{\text{fall}}$ (Zajac, 1989). Here, *f*(*s*, *k*) denotes the generated muscle excitation signal using e.g., the proposed DMPSynergies. The actual muscle activation is denoted by *a*, which is a function of the rise time constant τ_rise_ and the fall time constant τ_fall_. For our evaluations we implemented τ_rise_ = 10 ms and τ_fall_ = 40 ms (Winters and Stark, 1985).

**Muscle tendon dynamics** describe the complex and non-linear force generation properties of muscles. For an approximation a variety of models exist (Zajac, 1989). In these models a muscle is approximated by a number of musculotendinous units, each of which is implemented by a Hill-type contractile element in series with tendon. Characteristic properties of muscles are the optimal fiber length *L*^*M*^_0_, the maximum isometric force *F*^*M*^_0_, and the muscle pennation angle α, which are shown in Table A5 in the appendix for the investigated model of a human arm. The tendon dynamics in this musculoskeletal model were approximated by the muscle model proposed in Schutte et al. (1993).

**Musculoskeletal geometry** represents the path of a muscle from its origin to its insertion that can be implemented as a series of straight-line path segments, which pass through a series of via points (Delp et al., 2007). To simulate how muscles wrap over underlying bone and musculature wrapping surfaces i.e., cylinders, spheres and ellipsoids are implemented, where this model is based on the upper extremity model discussed in Holzbaur et al. (2005). A detailed description of the implemented musculoskeletal geometry is given in the supplement (in form of a simulation model file,.osim).

**Multibody dynamics** are simulated by the physics simulation application OpenSim (Delp et al., 2007; Seth et al., 2011). It is an open source software that already implements a variety of muscle models (Zajac, 1989) and a large number musculoskeletal models are freely available. In our experiments the computational time needed to simulate a movement with a duration of e.g., 500 ms takes between 10 and 20 s (OpenSim implements numerical integrators with an adaptive time step) on a standard computer (3 GHz and 4 GB memory). However, we exploited parallel computing techniques for policy search, which resulted in a gain of factor 10. Alternatively, the muscle dynamics could be approximated via regression methods to speed-up the simulations (Chadwick et al., 2009).

### 2.4. Learning with movement primitives

**Figure 3:**
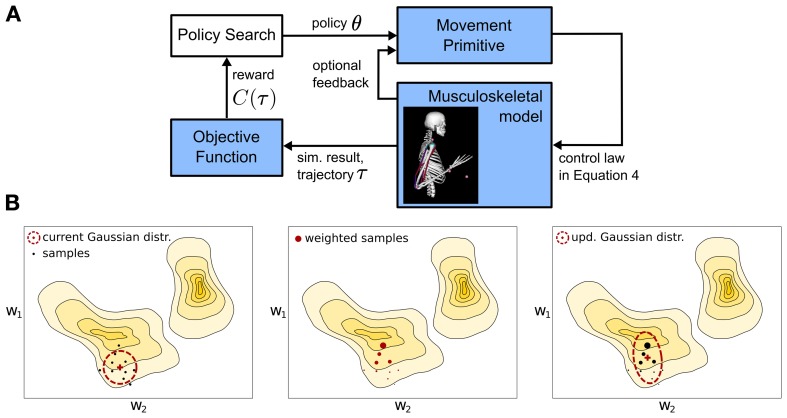
**Overview of the learning framework. (A)** A parametrized policy **θ** modulates the output of a movement primitive that is used to generate a movement trajectory τ. The quality of the movement trajectory is indicated by a sparse reward signal *C*(τ) which is used for policy search to improve the parameters of the movement primitive. For a single iteration the implemented policy search method - Covariance Matrix Adaptation (CMA) (Hansen et al., 2003) is sketched in **(B)**. The parameter space is approximated using a multivariate Gaussian distribution denoted by the ellipses, which is updated (from left to right) using second order statistics of roll-outs or samples that are denoted by the large dots (see text for details).

We denote the parametrization of a movement primitive by a policy vector **θ**. A widely used approach in robotics to learn these parameters is episodic reinforcement learning (Kober and Peters, 2011), which is outlined in Figure 3A. A policy search method is used to improve the movement primitive's representation **θ** assuming a given objective or reward function *C*(τ) ∈ ℝ^1^. Throughout this manuscript *C*(τ) denotes a cost value that is equivalent to the negative reward in classical reinforcement learning (Sutton and Barto, 1998). It indicates the quality of an executed movement. A trajectory τ = 〈**y**_1:*T*_, **u**_1:*T* − 1_ 〉 is specified by the simulated joint angles **y** and the applied controls (torques) **u**, where *T* denotes the number of time steps. We want to find a movement primitive's parameter vector θ^*^ = argmin_θ_*J*(θ) which minimizes the expected costs J(θ)=E[C(τ)|θ]. We assume that we can evaluate the expected costs *J*(θ) for a given parameter vector θ by performing roll-outs (samples) on the real or simulated system. In other words each movement trajectory is quantified by a single scalar reward *C*(τ), which can be used by an optimization method to improve the best guess of the movement policy θ.

For learning or optimizing the policy parameters θ a variety of policy search algorithms exist in the motor control literature. Examples are the REINFORCE (Williams, 1992), the episodic Natural Actor Critic (Peters and Schaal, 2008), the Power (Kober and Peters, 2011) or the *PI*^2^ (Theodorou et al., 2010) algorithm, which are reviewed in Kober and Peters (2011). Alternatively, standard optimization tools such as the 2nd order stochastic search methods (Hansen et al., 2003; Wierstra et al., 2008; Sehnke et al., 2010) can be used for policy search. These machine learning tools make no assumptions on a specific form of a policy and typically have just a single parameter to tune, the initial exploration rate. We therefore use the stochastic search method Covariance Matrix Adaptation (CMA) (Hansen et al., 2003) for learning the policy parameters in our experiments.

Roughly, CMA is an iterative procedure that locally approximates the function *C*(τ(θ)) by a multivariate Gaussian distribution, which is denoted by the ellipse in the sketch in Figure 3B. From left to right a single optimization step for a two-dimensional policy vector **θ** = [*w*_1_, *w*_2_] is shown. The colored regions denote the unknown optimization landscape, where solid lines depict equal *C*(τ) values. From the current Gaussian distribution, denoted by the ellipse in the left panel, CMA generates a number of samples, denoted by the black dots, evaluates the samples (the size of the dots in the center panel is proportional to their *C*(τ) values), computes second order statistics of those samples that reduced *C*(τ) and uses these to update the Gaussian search distribution, which is shown in right panel. For algorithmic details we refer to Hansen et al. (2003).

Note that for most interesting robotic tasks the unknown optimization landscape that is also sketched in Figure 3B is multi-modal and policy search might converge to a local optimum. Thus, the result of learning is sensitive to the initial policy parameters θ and for evaluating the convergence rate of different policy search methods multiple initial configurations should be considered (Kober and Peters, 2011). However, in this manuscript we evaluate the characteristics of movement primitive representations and put less emphasis on a particular policy search method. As we will demonstrate in our experiments CMA is robust in terms of converging to “good” solutions given the initial values of the evaluated movement primitive representations listed in the appendix.

In our experiments we compare single task learning with DMPs to learning multiple tasks simultaneously with DMPSynergies. With DMPs for each task *k* = 1..*K* an individual policy vector **θ**_*k*_ is learned, where the objective function used in policy search takes the task index as additional argument, i.e., *C*(τ, *k*). For learning multiple tasks simultaneously with DMPSynergies the policy vector **θ** encodes all *K* task specific parameters β_*m*, *k*_ and Δ*s*_*m*, *k*_, and all shared parameters denoted by θ_*m*_ in Equation 5 or Equation 6. The objective function is the sum of the individual task dependent costs *C*(τ) = ∑^*K*^_*k* = 1_ *C*(τ, *k*).

## 3. Results

We evaluated the proposed movement representation, the DMPSynergies, with simulations using three multi-task learning scenarios. A simple via-point task was used to illustrate the characteristics of the proposed movement representation. A challenging robotic learning task was used to generate rhythmic walking patterns for multiple step heights. Discrete reaching movements were learned using a musculoskeletal model of a human arm with eleven muscles.

### 3.1. Via-point reaching task with a simple toy model

**Figure 4:**
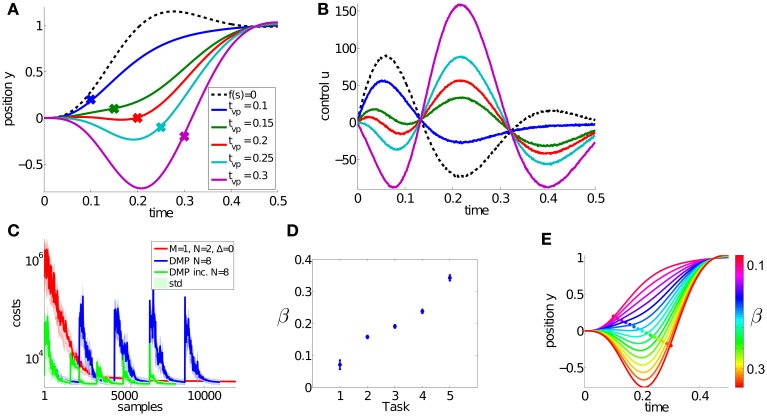
**Results for the dynamic via-point task.** The goal of this simple multi-task learning problem is to pass through five via-points, denoted by the large dots in **(A)** and navigate to the target state at 1. The corresponding controls (accelerations) of this dynamical system are shown in **(B)**. These five trajectories are simultaneously learned using DMPSynergies with a single synergy (*M* = 1) represented by *N* = 2 Gaussians. We compare to dynamic movement primitives (DMPs) with *N* = 8 Gaussians and to an incremental variant of DMPs in **(C)**. For the DMP approaches each task (via-point) has to be learned separately. Thus, the two learning curves have five peaks. In contrast with DMPSynergies we could learn these five tasks at once, which resulted in faster overall convergence. The plot in **(D)** illustrates the mean and the standard deviation of the learned β values for the DMPSynergy approach. Via interpolating β and by reusing the learned synergy new motor skills can be generated without re-learning. This is illustrated in **(E)**, where β ∈ [0.07, 0.34].

The goal of this simple multi-task learning problem is to pass through *k* = 1..5 via-points (*vp*_*k*_ ∈ {0.2, 0.1, 0, −0.1, −0.2}), denoted by the large dots in Figure 4A and navigate to the goal state *g* at 1. We used a point mass system (1 kg), where the state at time *t* is given by the position *y*_*t*_ and the velocity ${\dot{y}}_{t}$. The applied controls *u*_*t*_ shown in Figure 4B are computed using the linear feedback control law with *k*_*pos*_ = 400 and *k*_*vel*_ = 15 specified in Equation 4. The finite time horizon is given by *T* = 50. For the dynamical system in Equation 1 we used the parameters α_*z*_ = 2, β_*z*_ = 0.9 and τ = 0.1. Further parameter settings used for policy search are summarized in Table A1 in the appendix.

This task is specified by the objective function

$$C(k)=10^{5}{\left(y_{t_{{\text{vp}}_{k}}}-{\text{vp}}_{k}\right)}^{2}+10^{4}\left({\dot{y}}_{T}{}^{2}+10{\left(y_{T}-g\right)}^{2}\right)+5\cdot 10^{-4}\sum_{t=1}^{T}u_{t}.$$

The first two terms punish deviations from the via-point vp_*k*_ and the goal state *g*, where *y*_*t*_*vp*_*k*___ denotes the position of the state at the time index of the *k*th via-point. The last term punishes high energy consumption, where *u*_*t*_ denotes the applied acceleration. Note that for simplicity we did not introduce the variable τ denoting the movement trajectory in *C*(τ, *k*) in Subsection 2.4. We always add a Gaussian noise term with a standard deviation of σ = 0.5 to the control action to simulate motor noise.

We used a single synergy (*M* = 1) with *N* = 2 Gaussians to model the shared prior knowledge. The learning curve is shown in Figure 4C, where we compare to single-task learning using DMPs with *N* = 8 Gaussian basis functions. For the via-point task 8 Gaussians were optimal with respect to the convergence rate, where we evaluated representations using *N* = 2..20 Gaussians (not shown). Additionally, we compare to an incremental learning setup (DMP inc.) in Figure 4C, where the DMP representation is always initialized with the best learned solution from the previous task. On the x-axis the number of samples or trajectory evaluations on the point mass system is plotted. As it can be seen the proposed approach can benefit from the shared knowledge and has a faster overall learning performance.

In this experiment, for each task we fixed the time-shift Δ*s*_*k*_ = 0 and only learned the *k* = 1..5 weights β_*k*_ in Equation 5 (Note that the synergy index *m* was omitted as only a single synergy was used). For each of the *N* = 2 Gaussians we learned the mean μ, the bandwidth *h* and the amplitude *a* in Equation 7. Thus, in total 5 + 2 × 3 = 11 parameters were learned. In contrast with DMPs 8 Gaussian amplitudes were optimized.

The β values of the DMPSynergies representation for the five via-points are shown in Figure 4D for 10 runs. New motor skills can be generated without re-learning via a simple linear interpolation. The resulting trajectories are shown in Figure 4E. However, this is only the case in the simple via-point task. For more complex tasks these β values have to be learned.

### 3.2. Dynamic biped walker task

**Figure 5:**
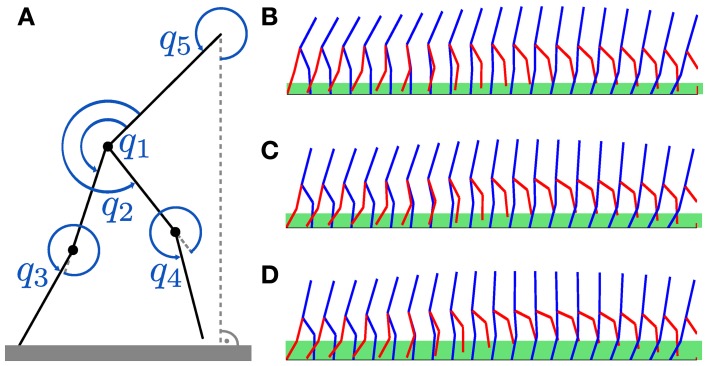
**Dynamic biped walker model. (A)** For the walker model, only the hip angles q_1_, q_2_ and the knee angles q_3_, q_4_ are actuated. The reference angle to the flat ground is denoted by q_5_. In this multi-task learning experiment we want to learn walking patterns for different step heights. Examples for step heights of 0.15, 0.25, and 0.3 m for a single step are shown in **(B–D)**. These patterns were learned using the proposed movement primitives with shared synergies (*M* = 2 and *N* = 3). The green bars in **(B–D)** denote the true (maximum) step heights, which are 0.19, 0.24, and 0.31 m.

To evaluate the DMPSynergies on a multi-dimensional robotic task we learned multiple walking patterns using a 5 degree-of-freedom (DoF) dynamic biped robot model, which is shown in Figure 5A. We demonstrate that by exploiting the shared knowledge among multiple walking gaits, solutions could be found more robustly and more efficiently in terms of learning speed compared to single task learning. Further, the shared synergies could be used to generalize new skills. The model is only as complex as required to study difficulties like limb coordination, effective underactuation, hybrid dynamics or static instabilities. More details on the design and challenges can be found in Westervelt et al. (2004).

The 10-dimensional state $q_{t}=[{\text{q}}_{1:5},{\dot{\text{q}}}_{1:5}]$ of the robot is given by the hip angles (*q*_1_ and *q*_2_), the knee angles (*q*_3_ and *q*_4_), a reference angle to the ground (*q*_5_), and the corresponding velocities ${\dot{\text{q}}}_{1:5}$. Only the hip and the knee angles are actuated. Thus, 4 dynamical systems in Equation 1 are used to generate desired trajectories for the linear feedback controller in Equation 4. A phase resetting strategy is implemented to facilitate learning (Nakanishi et al., 2004). At each impact of the swing leg the phase ϕ in Equation 8 is set to zero. This increases the stability of the robot as the gait cycle duration is implicitly given by the impact time.

The initial state **q**_1_ ∈ ℝ^10^, the goal state **g** and the control gains in Equation 4 were optimized in advance for a desired step height of r^*^ = 0.2 m to simplify learning. The resulting values are shown in Table A2 in the appendix. For rhythmic movements the goal state **g** ∈ ℝ^5^ models an attractor point which is only specified for joint angles and not for velocities in Equation 1. As for the via-point reaching task, Gaussian noise with σ = 1 is added to the simulated controls. For Equation 1 we used the parameters α_*z*_ = 2, β_*z*_ = 0.5 and τ = 0.06. The initial parameter values and the applied ranges used for policy search are shown in Table A3 in the appendix.

In this multi-task learning experiment we want to learn walking patterns for different desired step heights: r^*^_k_ ∈ {0.15, 0.2, 0.25, 0.3} m. Example patterns for step heights of 0.15, 0.25 and 0.3 m are shown in Figures 5B–D, where the green bars denote the maximum step heights during a single step (0.19, 0.24 and 0.31 m).

The objective function for a single walking task is given by the distance travelled in the sagittal plane, the duration of the simulation and deviations from the desired step height r^*^_k_ with *k* = 1..4:

(11) $$C(k)=-0.6(x_{T}-x_{1})+0.2(5-T\cdot \Delta t)+50\sum_{i=\;1}^{S}{\left(r_{i}-r_{k}^{*}\right)}^{2},$$

where *x* denotes the x-coordinate of the hip, *S* the number of steps and *r*_*i*_ the maximal step height during the *i*th step. We used a time step Δ*t* = 2 ms. The time horizon *T* ∈ [1, 5000] is given by the last valid state of the robot, where the biped does not violate the joint angle constraints specified by **q**_min_ and **q**_max_ in Table A2 in the appendix.

**Figure 6:**
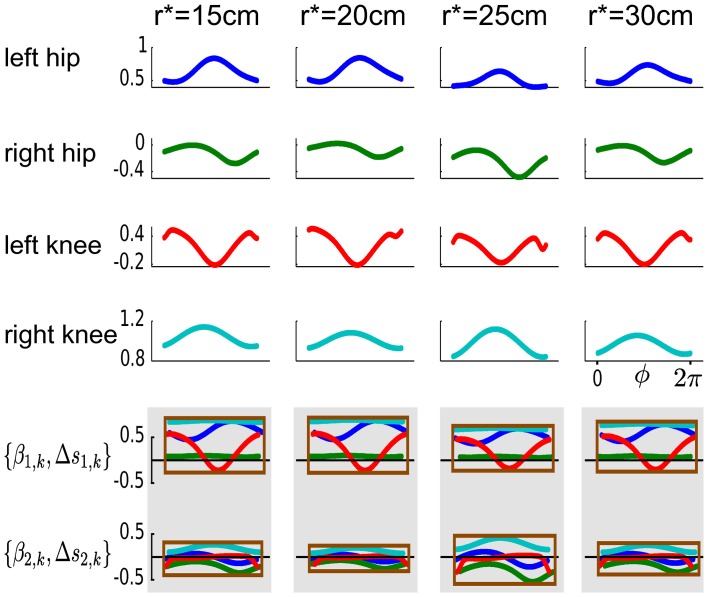
**Learned non-linear functions *f*(ϕ, *k*) for the walker task.** The learned non-linear functions *f*(ϕ, *k*) are illustrated in the first four rows. The four task instances, i.e., the desired step heights are shown from left to right. For each actuator (left hip, right hip, left knee, and right knee) an individual function *f*(ϕ, *k*) is used that is generated by combining two learned synergies shown in the last two rows. These synergies are shared among multiple task instances and can be scaled and shifted in time (via β_*m*, *k*_ and Δ*s*_*m*, *k*_). This is indicated by the enclosing rectangles, where the color of the synergies is used to distinguish the four actuators of the walker model.

With the proposed DMPSynergies the non-linear function *f*(ϕ, *k*) in Equation 6 is generated by combining a set of learned synergies that are shared among multiple task instances, i.e., the four (*k* = 1..4) desired step heights. This combination mechanism is illustrated for a representation using *M* = 2 synergies modeled by *N* = 3 Gaussians in Figure 6. For each actuator (left hip, right hip, left knee, and right knee) an individual function *f*(ϕ, *k*) is generated, which is subsequently used to modulate an attractor system shown in Equation 1 to compute the desired movement trajectories. The shared synergies shown in the last two rows in Figure 6 can be scaled and shifted in time. This is indicated by the enclosing rectangles. Note that the color of the synergies is used to distinguish the four actuators of the walker model.

**Figure 7:**
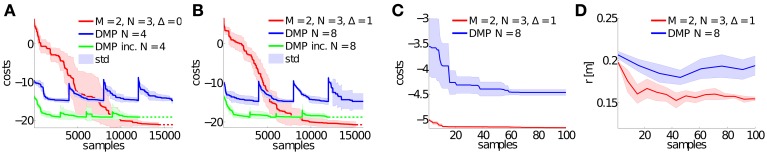
**Learning curves for the biped walker task.** This figure illustrates the learning performance over 10 runs of the proposed approach using *M* = 2 synergies with *N* = 3 Gaussian basis functions. In **(A)** the time-shift variables Δ**s** are not learned and set to zero. Whereas, in **(B)** also these Δ**s** variables are adapted during learning. We compare to the dynamic movement primitives (DMP) with *N* = 4 Gaussians in **(A)** and to DMPs with *N* = 8 Gaussians in **(B)**. *DMP inc.* denotes an incremental learning setup, where DMPs were initialized with the best result from the previous task. Generalizing to a new step height (r^*^ = 0.1 m) is shown in **(C)**, where we applied the best learned policy for DMPSynergies from **(B)** and only optimized the weights β_1:2_ for the two (fixed) synergies. The corresponding average step height over all steps is shown in **(D)**. We compare to DMPs with *N* = 8 Gaussians.

We evaluated different movement primitive representations with increasing complexity compared to single-task learning using DMPs with *N* = 4 and *N* = 8 Gaussians. The average final costs C_mean_ after learning over 10 runs are shown in Table 1. In the most simple representation we used *M* = 2 synergies modeled by *N* = 2 Gaussians. More complex representations implementing time-varying synergies are denoted by the symbol Δ = 1 in Table 1. Here, additionally the time-shifts Δ*s*_1:*M*_ were learned for all synergies and all actuators. However, the final learning performance did not outperform the representation with fixed time-shifts (i.e., *M* = 2, *N* = 3 and Δ = 0: −21.4 ± 0.4 compared to *M* = 2, *N* = 3 and Δ = 1: −20.5 ± 1.4). This can be also seen in Figure 7, where we plot the learning curve for synergies with Δ*s*_1:*M*_ = 0 in Figure 7A and the results for time-varying synergies in Figure 7B.

**Table 1:**
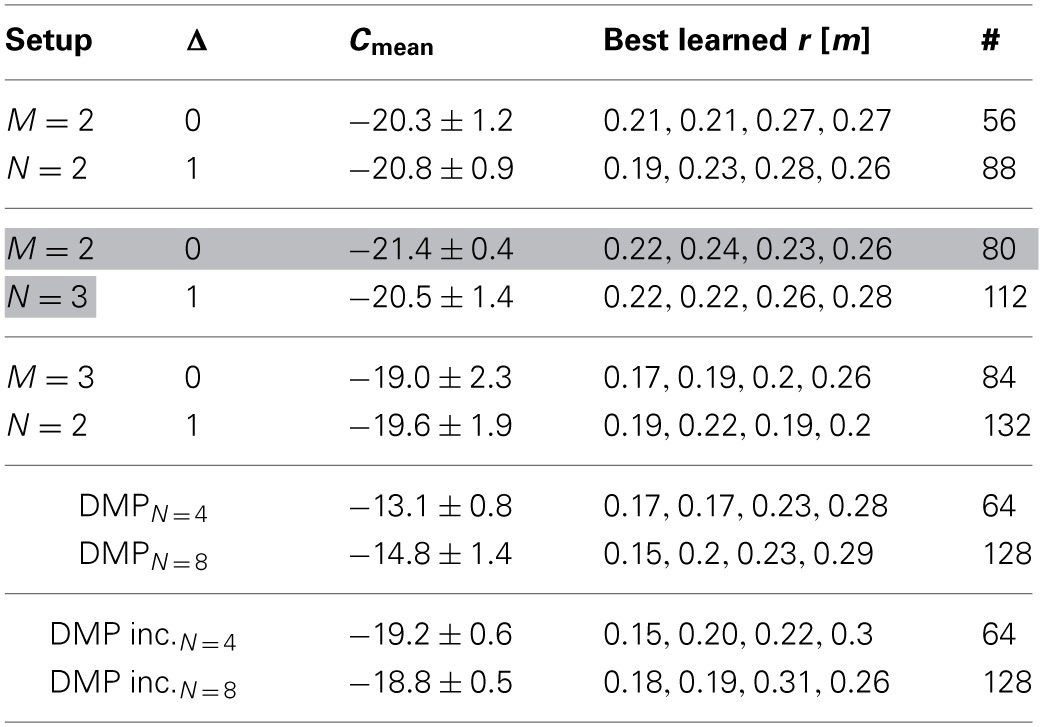
**Achieved costs for the walker task, where the standard deviation is denoted by the symbol ±**.

In the second column the symbol Δ denotes if additionally 16 M time-shift variables Δs are learned. The total number of parameters is denoted by the symbol #, where e.g., for the representation in the 1st row 4 M = 8 task related weights and 3 · M · N · 4 = 48 shared parameters were optimized. The best results with the lowest costs denoted by C_mean_ are highlighted in gray shading.

The average final cost value of the DMP representation is higher (i.e., DMP_*N* = 8_: −14.8 ± 1.4) compared to the best costs achieved with shared synergies (*M* = 2, *N* = 3 and Δ = 0: −21.4 ± 0.4). This holds also for an incremental learning setup (e.g., DMP inc._*N* = 4_: −19.2 ± 0.6), where DMPs were initialized with the best result from the previous task.

**Figure 8:**
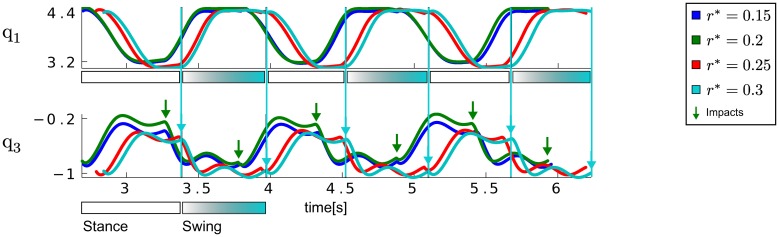
**Results for the biped walker task.** This figure illustrates the (initially) left hip angle denoted by q_1_ and the left knee angle (q_3_) for the multi-task learning scenario. Shown are the best learned trajectories using the proposed approach (with *M* = 2, *N* = 3 and Δ = 1) for the desired step heights of r^*^ ∈ {0.15, 0.2, 0.25, 0.3}. The true step heights of the learned walking patterns are 0.22 ± 0.07, 0.22 ± 0.08, 0.26 ± 0.08, 0.28 ± 0.08. The points in time of the ground contacts are denoted by large arrows for desired step heights of 0.25 m and 0.3 m. For the later additionally the duration of the stance and the swing phases are illustrated by large boxes.

The joint angle trajectories of the left hip and knee joint for the DMPSynergy representation using *M* = 2 synergies modeled by *N* = 3 Gaussians and Δ = 1 are illustrated in Figure 8. The average step heights were *r* ∈ {0.22, 0.22, 0.26, 0.28}, which do not match the desired step heights r^*^ ∈ {0.15, 0.2, 0.25, 0.3}. The reason for this is that the objective function in Equation 11 is designed to prefer correct multi-step walking movements over exact matches of the step heights since learning to walk is already a complex learning problem (approximately 90% of the costs are determined by the travelled distance and only 5% are caused by the distance to the desired step heights). However, for the different desired step heights the shape of the trajectories as well as the moment of the impact vary. The moments of impact are denoted by arrows in Figure 8.

While generalizing to new motor skills was straightforward for the simple via-point task, for the walking tasks a linear interpolation turns out to be ineffective. We therefore demonstrate in Figures 7C,D how a new walking pattern for a desired step height of r^*^ = 0.1 m can be learned be reusing the previously learned prior knowledge (taking the best solution for r^*^ = 0.25 m) for *M* = 2 synergies modeled by *N* = 3 Gaussians and Δ = 1. Only the weights β_1:*M*_ are optimized in this experiment, keeping the learned time-shifts fixed. The costs in Figure 7C and the average step height *r* in Figure 7D demonstrate the advantage of using a fixed prior, where we compare to DMPs with *N* = 8 Gaussians.

### 3.3. Multi-directional reaching task with a musculoskeletal model of the human arm

**Figure 9:**
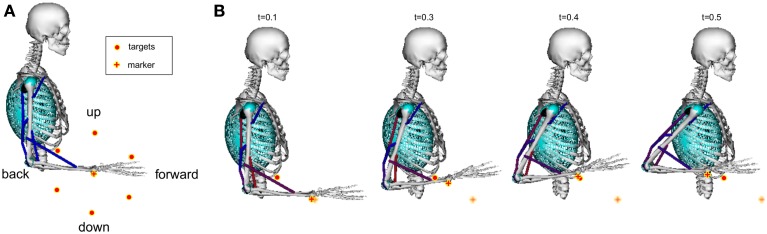
**Musculoskeletal model for learning reaching tasks.** A model of a human arm with eleven muscles shown in Table A5 in the appendix was used to learn six reaching skills in the sagittal plane **(A)**. As reward signal we encoded the distance to a marker placed on the radial stylion (denoted by the *plus* symbol) and punished large muscle excitation signals. Targets are denoted by large dots. We focused on fast reaching skills of 500 ms duration, where an example movement is shown in **(B)**. To simulate how muscles wrap over underlying bone and musculature wrapping surfaces are implemented as cylinders, spheres and ellipsoids (Holzbaur et al., 2005).

A simplified model of a human arm based on the model by Holzbaur et al. (2005) was used to learn six reaching tasks simultaneously. The shoulder and the elbow joint were modeled by hinge joints. Thus, only movements in the sagittal plane were possible. The initial arm configuration and the six target locations (with a distance of 15cm to a marker placed on the radial stylion) are shown in Figure 9A. A learned example movement is illustrated in Figure 9B, where the cylinders, the spheres and the ellipsoids denote wrapping surfaces discussed in Subsection 2.3.

We focused on fast reaching movements of 500 ms duration (*T* = 500 and Δ*t* = 1 ms) that can be implemented in an open-loop control scheme. Note that with our approach also closed-loop systems with feedback could be implemented, as discussed below. Thus, the learnable non-linear function **f**(*s*, *k*) in Equation 10 is directly used as input to the system in form of muscle excitation patterns. The parameter settings for learning are shown in Table A4 in the appendix.

For learning the reaching tasks we evaluated the Euclidean distance of a marker **v**_*k*_(*t*) placed on the radial stylion to a given target **g**_*k*_, where *k* = 1..6 denotes the task index. Additionally, large muscle excitations signals are punished:

(12) $$C(k)=\sum_{k=1}^{6}3\cdot \frac{1}{T}\sum_{t=1}^{T}\|g_{k}-v_{k}(t)\|+10^{-3}\int_{s=0}^{1}f{\left(s,k\right)}^{T}f(s,k)\text{d}s,$$

where ‖.‖ denotes the Euclidean distance between the marker **v**_*k*_(*t*) and the target **g**_*k*_ at time *t*.

We evaluated five movement representations, defined in Equation 10, with an increasing number of shared synergies, i.e., *M* = {1, 2, 3, 4, 5}. Each synergy is represented by a single (*N* = 1) Gaussian. For each target and for each synergy the task-specific parameters β_*k*, *m*_ and Δ*s*_*k*, *m*_ are learned. The number of task-specific and the number of task-invariant or shared parameters is shown in Table 2.

**Table 2:** **Details of the evaluated parametrizations and achieved costs for the reaching task**.

**No. of synergies**	***C*_mean_**	**#_*K*_**	**#_*M*_**	**Total**
*M* = 1	2.38 ± 0.05	12	33	45
*M* = 2	1.52 ± 0.12	24	66	90
*M* = 3	1.15 ± 0.12	36	99	135
*M* = 4	1.15 ± 0.05	48	132	180
*M* = 5	1.17 ± 0.05	60	165	225

*M* denotes the number of implemented synergies, C_mean_ the final cost values, and the symbol ± the standard deviation. We use #_K_ to denote the number of task-specific parameters (*6* · M) and #_M_ to denote the number of task-invariant or shared parameters (3 · 11 · M).

**Figure 10:**
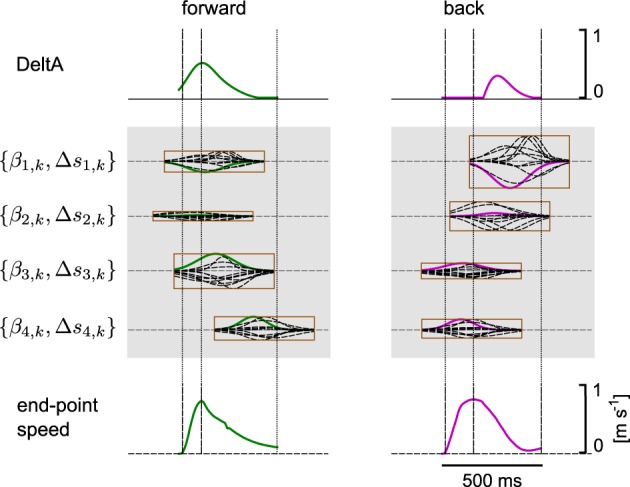
**Synergy combination mechanism.** We hypothesize that a muscle excitation signal can be generated by combining a small number of learned synergies. Here, we illustrate this combination process for the deltoid anterior (DeltA) with four synergies for two movement directions. For the two movement directions different combination coefficients β_*m*, *k*_ and different time-shift parameters Δ*s*_*m*, *k*_ were learned. The synergies are represented by a single parametrized Gaussian, where the corresponding basis function for DeltA is denoted by a bold line in the enclosing rectangles.

**Figure 11:**
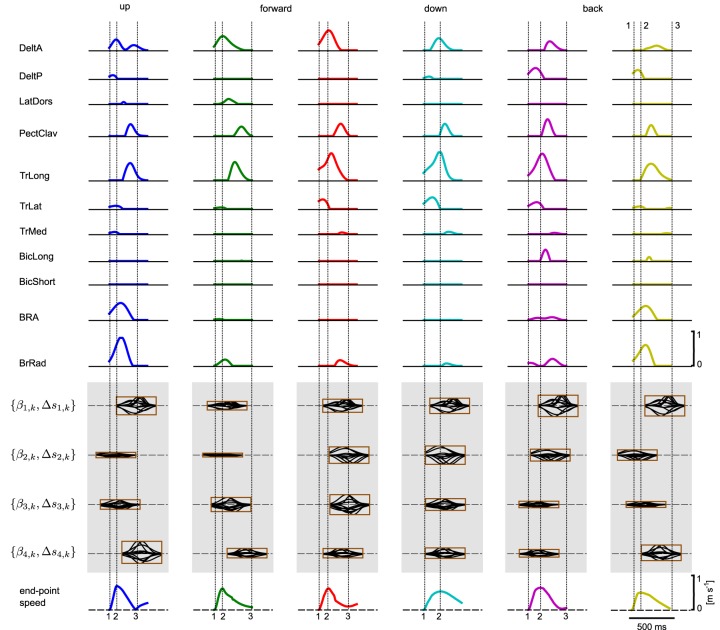
**Learned muscle excitation patterns.** Shown are the muscle excitation patterns for all six targets (from left to right) and for all muscles (rows one to eleven). In the last row the tangential velocity profiles of the marker placed on the radial stylion is illustrated (see text for details).

We hypothesized that a muscle excitation signal can be generated by combining a small number of learned synergies. An example for the anterior deltoid muscle (DeltA) is shown in Figure 10 for two movement directions. Here, DMPSynergies with *M* = 4 synergies were used to generate the muscle excitation patterns. The muscle excitation patterns for all six movement directions and all eleven muscles are shown in Figure 11. Two observations can be made: first, as our objective function in Equation 12 punishes large muscle excitation signals a sparse representation of multiple motor skills is learned. Second, the learned muscle patterns partially show the typical triphasic behavior of human movement (Angel, 1975; Hallett et al., 1975; Berardelli et al., 1996; Chiovetto et al., 2010), where individual muscles (e.g., DeltA, PectClav and BRA in the first column in Figure 11) become activated at the onset of the movement, shortly before the velocity peak to decelerate, and finally, multiple muscles co-contract at the target location. These three events are denoted by the labels 1, 2, and 3 in the last row in Figure 11, where a threshold of 2 cm s^−1^ was used to determine the movement onset and the termination of the movement.

**Figure 12:**
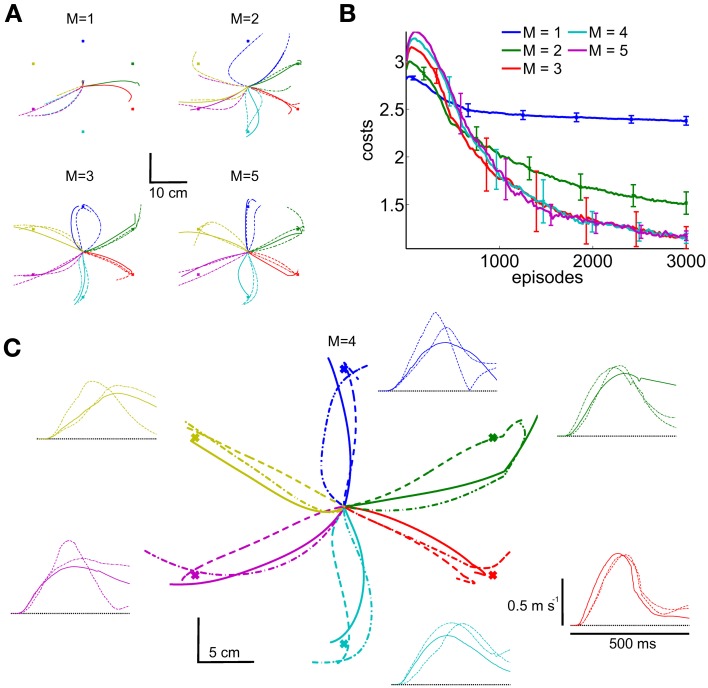
**Learning multi-directional reaching movements.** We evaluated five movement representations with an increasing number of shared synergies, i.e., *M* = {1, 2, 3, 4, 5}. The resulting trajectories of the marker placed on the radial stylion are shown in **(A,C)**, where with less than three synergies not all targets can be reached. Illustrated are three independent learning results. In **(B)** we illustrate the average learning curves over 10 runs for these movement representations. For the representation using *M* = 4 synergies shown in **(C)** additionally the tangential velocity profiles are illustrated.

Comparing all five movement representations (*M* = {1, 2, 3, 4, 5}), we found that at least three synergies were necessary to accomplish all reaching tasks. This is shown in Figure 12A, where with only one (*M* = 1) or two synergies (*M* = 2) not all targets can be reached. Shown are the marker trajectories of three independent learning sessions (out of ten runs). Note that similar findings were obtained in analyzing human arm reaching movements, where four to five synergies were observed (d'Avella et al., 2006). The corresponding learning curves for all five movement representations are shown in Figure 12B, where the parametrizations with *M* = 3..5 synergies perform equal. This is also reflected in the final costs shown in Table 2 (rows 3..5). As an example the marker trajectories and the tangential velocity profiles for the representation using *M* = 4 synergies are illustrated in Figure 12C. As we evaluated an open-loop control scheme these marker trajectories did not exactly terminate at the target location (after the limited number of episodes for learning). However, by increasing the number of episodes or by adding feedback the terminal accuracy could be improved.

**Figure 13:**
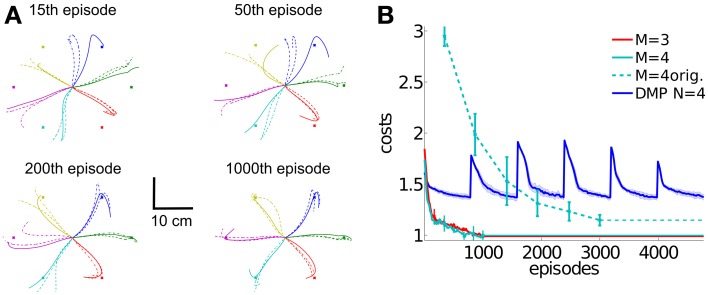
**Generalization to new reaching directions.** For testing the generalization ability of the proposed DMPSynergies we fix the learned shared synergies and only adapt the task-specific parameters, i.e., the mixing coefficients β_*m*, *k*_ and the time-shift parameters Δ*s*_*m*, *k*_. The *K* = 6 targets were rotated by 30 degrees, where in **(A)** the marker trajectories after 15, 50, 200, and 1000 episodes for a movement representation with *M* = 4 synergies are shown. In **(B)** we show the averaged learning curves for DMPSynergies with three and four synergies over 10 runs (*M* = 3 and *M* = 4). The learning curve for the unperturbed scenario from the previous experiment is denoted by the dashed line (*M* = 4 orig.). For a comparison the blue line denoted by DMP *N* = 4 illustrates the convergence rate of single task learning.

For testing the generalization ability of DMPSynergies we rotated all six targets by 30 degrees and only re-learned the task-specific coefficients, i.e., the mixing coefficients β_*m*, *k*_ and the time-shift parameters Δ*s*_*m*, *k*_. Interim solutions with a movement representation implementing *M* = 4 synergies are shown in Figure 13A. Note that, as we evaluated an open-loop controller, the rotated targets were unknown to the controller. Solely the objective function in Equation 12 quantifies deviations from the targets. After 15 episodes a first trend toward the new targets was visible, however, most of the trajectories (three learned solutions are illustrated) ended at the original targets. The corresponding learning curves for DMPSynergies with three (*M* = 3) and four (*M* = 4) synergies are shown in Figure 13B. The learning curve for the unperturbed scenario from the previous experiment is denoted by the dashed line (*M* = 4 orig.). Note that in both - the unperturbed and the perturbed experiments *K* = 6 reaching movements were learned, which demonstrates the benefit of the shared learned knowledge when generalizing new skills. For a comparison the blue line denoted by DMP *N* = 4 illustrates the convergence rate of single task learning with DMPs, where DMPSynergies (*M* = 4 orig.) can compete in terms of learning speed.

## 4. Discussion

We proposed a movement representation based on learned parametrized synergies (DMPSynergies) that can be linearly combined and shifted in time. These learned synergies are shared among multiple task instances significantly facilitating learning of motor control policies. This was demonstrated on simulated robotic and on musculoskeletal systems. Below we discuss the significance and the implication of our findings with respect to robotics and biological motor control.

### 4.1. Exploiting shared synergies for motor skill learning in robotics

For motor skill learning in robotics a common strategy is to use parametrized elementary movements or movement primitives (Kober and Peters, 2011). In this paper we proposed a generalization of the most widely used movement primitive representation in robotics, dynamic movement primitives (DMPs) (Schaal et al., 2003; Ijspeert et al., 2013). DMPs evaluate parametrized dynamical systems to generate trajectories. The dynamical system is constructed such that the system is stable. This movement representation has many advantages. It is a model-free approach, partially explaining its popularity in robotics as model learning in high-dimensional stochastic robotics systems is challenging. Further, its stable attractor system facilitates learning and DMPs can represent both rhythmic and discrete movements. Meta parameters can be used for adapting the movement speed or the goal state. Finally, the movement representation depends linearly on the policy parametrization, i.e., the learnable function *f* depends linearly on the parameters θ of the movement primitive: *f*(*s*) = Φ(*s*)^*T*^ θ, where *s* is the time or phase variable. As a result, imitation learning for DMPs is straightforward, as this can simply be done by performing linear regression (Schaal et al., 2003). However, for each task *k* an individual set of parameters θ_*k*_ has to be learned, which unnecessarily complicates learning of a large number of related motor skills. In contrast we proposed a generalization that allows for reusing shared knowledge among multiple related motor skills, i.e., the parameter vector θ is task-invariant.

In particular, we replaced the non-linear modulation function *f*(.) in DMPs by a hierarchical function approximator. On the lower level task related parameters (amplitude scaling weights and time-shift parameters) are used to modulate a linear superposition of basis functions. These basis functions encode shared higher level knowledge and are modeled by a mixture of Gaussians. With the proposed DMPSynergies representation discrete and rhythmic movements can be generated. By using Gaussians at the higher level DMPs can be implemented as special case. However, the DMPSynergies can compete with DMPs in terms of learning efficiency while allowing for learning multiple motor skills simultaneously.

This was demonstrated in two robotic multi-task learning scenarios, where we showed that, with the DMPSynergies, good policies could be found more reliably, i.e., local minima with high cost values were more often avoided, more efficiently (fewer samples were needed), and new skills could be generalized by exploiting the previously learned shared knowledge. A simple via-point task was used to demonstrate the characteristics of the approach, where the proposed movement representation could be used to generalize new movement trajectories by applying a linear interpolation on the synergy's weights β. In a second robotic task, a biped walker task, the hierarchical representation was used to learn walking patterns with multiple step heights. In this complex reinforcement learning task, it was shown that better solutions were found more reliably by exploiting the learned shared knowledge, which is a strong feature of a movement representation. While also with the classical DMP approach high quality movement gaits were learned, on average the achieved costs were higher compared to the proposed hierarchical synergies representation, i.e., −19.2 ± 0.6 for DMPs with 4 Gaussians (and with incremental learning) compared to −21.4 ± 0.4 when using *M* = 2 synergies with *N* = 3 Gaussians (where the time-shift parameters were fixed and set to zero, Δ = 0). In this experiment 10, 000 samples were needed to learn 4 walking gaits simultaneously, where the DMPSynergies approach can compete with DMPs (15, 000 samples). Additionally, we demonstrated in a generalization experiment that walking patterns for an unknown step height (r^*^ = 0.1 m) could be learned with 100 samples by exploiting the previously learned prior knowledge.

While DMPs (Schaal et al., 2003; Ijspeert et al., 2013) are most closely related to our shared synergies approach, there exist a few other approaches (Chhabra and Jacobs, 2006; Alessandro et al., 2012) also implement shared knowledge. In Chhabra and Jacobs (2006) a variant of non-negative matrix factorization (d'Avella et al., 2003) was used to compute the synergies given a set of trajectories created by applying stochastic optimal control methods (Li and Todorov, 2004). In Alessandro et al. (2012) an exploration phase was introduced to compute the dynamic responses of a robot system with random initialization. After a reduction phase, where a small number of proto-tasks were executed, a reduced set of dynamic responses was used to compute the synergies matrix by solving a linear system of equations. We proposed an alternative for learning the synergies and their combination parameters, where all unknowns are learned in a reinforcement learning setting from a single sparse reward signal. Moreover, for robotic tasks we embed the synergies approach in stable dynamical systems like in DMPs. This combines the benefits of DMPs and muscle synergies, namely the efficient learning ability of DMPs in high-dimensional systems and the hierarchical representation of movements that can be used for multi-task learning.

As with DMPs the complexity of the DMPSynergies representation can be scaled by the number of combined synergies or the number of implemented Gaussians modeling these synergies. However, as the trajectories generated with our representation depend non-linearly on the policy parameters (in contrast to DMPs) more sophisticated decomposition strategies like for example d'Avella and Tresch (2001); Chiovetto et al. (2013) are needed for imitation learning. With such approaches the extracted synergies could be implemented as initial solutions in our learning framework.

### 4.2. Learned shared synergies for biological movement generation

The idea of reusing shared knowledge for movement generation is a well-known concept in biological motor control. Muscle activation patterns recorded during multiple task instances of natural motor behavior, i.e., fast reaching movements of humans (d'Avella et al., 2006), primate grasping movements (Overduin et al., 2008), or walking patterns (Dominici et al., 2011), could be efficiently modeled by combining only few muscle activation patterns. In particular, time-varying muscle synergies (d'Avella et al., 2003; Bizzi et al., 2008) were proposed to be a compact representation of muscle activation patterns. The key idea of this approach is that muscle activation patterns are linear sums of simpler, elemental functions or synergies. Each muscle synergy can be shifted in time and scaled with a linear factor to construct a large variety of activation patterns. In this manuscript we proposed a generative model to represent and learn time-varying synergies (d'Avella et al., 2006).

The proposed framework allows for studying the concept of muscle synergies from a generative perspective in contrast to the analytical approach, where muscle synergies are identified from observed data. Applying such a generative approach to a musculoskeletal model, we could provide a proof-of-concept of the feasibility of a low-dimensional controller based on shared synergies and a demonstration of its learning efficiency. Moreover, we could ask different question, i.e., how does performance scale with the complexity of the movement representation, how sparse is the encoding of the muscle patterns to solve particular tasks, and how well does the learned representation generalize to new movements? We addressed these questions in a multi-directional reaching task, where we investigated a musculoskeletal model of the upper limb with 11 muscles. Motor skills for 6 reaching directions were learned within 3000 episodes and by exploiting the learned shared synergies movements for rotated target directions can be generalized 3 times faster (Figure 13). We found that a minimum of three synergies were necessary to solve the task (Figure 12B). In our objective function large muscle excitation signals were punished, which resulted in a sparse representation of muscle excitation patterns. This sparse representation illustrated in Figure 11 shows similarities to observed electromyographic activity recorded in related human reaching tasks (d'Avella et al., 2006), i.e., triphasic muscle patterns, where some of the muscles contributed at the movement onset, some at point of the maximum tangential velocity, and some at the end of the movement to co-contract. However, sensor feedback might be an important modulation signal to make this effect more pronounced.

The model was designed to capture salient features of human musculoskeletal system, such as muscle activation dynamics, Hill-type musculotendinous units, realistic geometry. However, to reduce the computational effort needed to simulate a movement we made a few simplifying assumptions. First, a limited number of muscles (11) were implemented, where simplified wrapping objects and muscle paths were modeled. Further, we implemented the shoulder and the elbow joint as hinge joints. Thus, only reaching movements in the sagittal plane could be performed. Finally, we focused on fast reaching movements in an open-loop control scheme. This was a valid assumption for comparing to human data for fast reaching movements (d'Avella et al., 2006). However, our proposed learning and control framework also allows for implementing closed-loop controllers, i.e., when introducing an inverse kinematics model Ψ ∈ ℝ^*Dx*3^ in Equation 1, i.e., $\tau \dot{z}=\Psi (\alpha_{z}(\beta_{z}(g-y^{*})-z))+f,$ where *D* denotes the number of actuators and we assumed that the goal state **g** lives in a three-dimensional Cartesian space. The inverse kinematics model Ψ maps the feedback error signal into the muscle pattern space and modulates the learned muscle excitation basis **f** ∈ ℝ^*D*^. With such closed-loop systems we might better understand the contribution of feedback to muscle control in biological movement generation (Lockhart and Ting, 2007).

Musculoskeletal models have been used before to investigate movement generation with muscle synergies (Berniker et al., 2009; Neptune et al., 2009; McKay and Ting, 2012). Berniker and colleagues used model-order reduction techniques to identify synergies as a low-dimensional representation of a non-linear system's input/output dynamics and optimal control to find the activations of these synergies necessary to produce a range of movements. They found that such a set of synergies was capable of producing effective control of reaching movements with a musculoskeletal model of a frog limb and that it was possible to build a relatively simple controller whose overall performance was close to that of the system's full-dimensional non-linear controller. Neptune and colleagues generated muscle-actuated forward dynamics simulations of normal walking using muscle synergies identified from human experimental data using non-negative matrix factorization as the muscle control inputs. The simulation indicated that a simple neural control strategy involving five muscle synergies was sufficient to perform the basic sub-tasks of walking. McKay and Ting, studying an unrestrained balance task in cats, used a static quadrupedal musculoskeletal model of standing balance to identify patterns of muscle activity that produced forces and moments at the center of mass (CoM) necessary to maintain balance in response to postural perturbations. CoM control could be accomplished with a small number of muscle synergies identified from experimental data, suggesting that muscle synergies can achieve similar kinetics to the optimal solution, but with increased control effort compared to individual muscle control. In line with these simulation studies, we also found that a small number of muscle synergies was sufficient to perform multiple reaching tasks in a forward dynamic simulation of a musculoskeletal model. However, we did not use experimental data or model-order reduction techniques to identify muscle synergies. In our framework, both synergy structural parameters and synergy combination parameters were found with reinforcement learning, supporting the generality of the solutions identified. Moreover, we were able to test the generalization ability of the synergies in the same framework by optimizing only the task-specific synergy combination parameters.

The proposed reinforcement learning framework with movement primitives relates to optimal control approaches in the biological motor control literature (Delp et al., 2007; Erdemir et al., 2007). In these simulation studies muscle patterns are parametrized by e.g., bang-bang (on-off) controls, constant control values, or control vectors approximated with polynomials [see Table 2 in Erdemir et al. (2007) for an overview of different control strategies]. However, to the best of our knowledge non of these approaches implemented shared synergies as control signal representation for learning multiple task instances simultaneously. Even with complex representations, e.g., with *M* = 5 synergies learning 225 parameters converged within 3000 episodes, which is a promising feature of the proposed approach for studies on more complex musculoskeletal models.

In this manuscript we demonstrated how time-varying synergies (d'Avella et al., 2006) can be implemented and learned from scratch. Interestingly, by adding an additional constraint on the movement representation, i.e., by using a single policy vector for all actuators anechoic mixing coefficients (Giese et al., 2009) can be implemented. However, in general any synergy representation such as synchronous synergies (Ivanenko et al., 2004; Dominici et al., 2011) used for locomotion can be learned. Thus, we do not argue for a particular synergy representation. Our representation was motivated to extend the widely used DMPs (Schaal et al., 2003) for exploiting shared task-invariant knowledge for motor skill learning in robotics.

## 5. Conclusion

We proposed a movement primitive representation implementing shared knowledge in form of learned synergies. The representation competes with the state-of-the-art, it can implement DMPs (Schaal et al., 2003) as a special case, and it allows for an efficient generalization to new skills. Importantly, shared knowledge simplifies policy search in high-dimensional spaces, which was demonstrated in a dynamic biped walking task. Further, the proposed learned synergies are a compact representation of high-dimensional muscle excitation patterns, which allows us to implement reinforcement learning in musculoskeletal systems. In such frameworks muscle patterns are learned from scratch using a sparse reward signal, where we could investigate how muscles and muscle synergies contribute to a specific task, how complex a task-invariant representation must be, and how well the learned synergies generalize to changes in the environment. In a multi-directional arm reaching experiment we provided first insights to these questions. In future research the proposed movement generation and learning framework will be used to study feedback signals and feedback delays, imitation learning from biological data and the effect of simulated muscle surgeries.

### Conflict of interest statement

The authors declare that the research was conducted in the absence of any commercial or financial relationships that could be construed as a potential conflict of interest.

## Appendix

For the via-point task the parameter settings for learning are shown in Table A1. Initial parameter values and parameter settings for policy search for the biped walker task are shown in Table A2 and in Table A3. In Table A4 we list the learning settings for the multi-directional reaching task using a musculoskeletal model of a human arm. The implemented muscles and their characteristic parameters are shown in Table A5.

**Table A1:** **Parameter settings for the discrete via-point task**.

**Parameter**	**Range**	**Initial value**
*a*, (*w*)	∈ [−5, 5]	*a*^0^ = 0
μ, (μ)	∈ [0, 1]	μ^0^ spaced linearly in [0, 1]
*h*, (*h*)	∈ [0.01, 1]	*h*^0^ = 0.1
β	∈ [0, 100]	β^0^ = 1

In brackets are the variable names for the dynamic movement primitives, which are used for comparison.

**Table A2:** **Biped walker setting of pre-optimized quantities**.

**Variable**	**Value**
*q*_1_	[3.5, 4.4, −0.07, −0.5, −0.7, −1.5, −0.6, −1.0, 0.3, −0.4]
*g*	[2.8, 4.5, −0.3, −1.8]
*k*_pos_	[632.5, 885.6, 463.4, 643.7]
*k*_vel_	[14.5, 13.2, 38.6, 40.6]
*q*_min_	[2.8, 2.8, −2.6, −2.6, −1.04]
*q*_max_	[4.7, 4.7, 0, 0, 1.0]

**Table A3:** **Policy search parameter settings for the rhythmic walking task**.

**Parameter**	**Range**	**Initial value**
*a*, (*w*)	∈ [−2, 2]	*a*^0^ = 0
μ, (μ)	∈ [0.8 μ^0^, 1.2 μ^0^]	μ^0^ spaced linearly in [0, 2π]
*h*, (*h*)	∈ [0.1, 10]	*h*^0^ = 1
β	∈ [0, 2]	β^0^ = 1
Δ*s*	∈ [0, 0.5]	Δ*s*^0^ = 0

In brackets are the variable names for the dynamic movement primitives.

**Table A4:** **Parameter settings for the multi-directional reaching task**.

**Parameter**	**Range**	**Initial value**
*a*	∈ [−1, 1]	*a*^0^ = 0.5
μ	∈ [0.3, 0.7]	μ^0^ spaced linearly in [0.3, 0.7]
*h*	∈ 8 [10^−5^, 10^−3^]	*h*^0^ = 8 · 10^−4^
β	∈ [0, 1]	β^0^ = 1
Δ*s*	∈ [−0.2, 0.2]	Δ*s*^0^ = 0

**Table A5:** **Characteristic muscle parameters of an upper limb model taken from Garner and Pandy (2001)**.

	***L*^*M*^_*0*_ [cm]**	***L*^*T*^_*s*_ [cm]**	***F*^*M*^_*0*_ [**N**]**	α **[rad]**
Anterior deltoid (DeltA)	14.68	**9.3** (1.64)	277.48	0
Posterior deltoid (DeltP)	17.02	5.93	567.15	0
Latissimus dorsi-thoracic (LatDors)	34.87	14.75	173.43	0
Pectoralis major-clav (PectClav)	22.65	0.45	342.46	0
Triceps brachii-long (TrLong)	15.24	**25.05** (19.05)	629.21	0.26
Triceps brachii-lateral (TrLat)	6.17	19.64	1268.87	0.26
Triceps brachii-medial (TrMed)	4.9	**18.19** (12.19)	619.67	0.26
Biceps-long (BicLong)	15.36	**32.93** (22.93)	392.91	0.17
Biceps-short (BicShort)	13.07	**26.98** (22.98)	461.76	0.17
Brachialis (BRA)	10.28	**9.75** (1.75)	853	0.17
Brachioradialis (BrRad)	27.03	6.04	101.58	0.08

The tendon slack length is denoted by L^T^_s_, the maximum isometric force by F^M^_0_, the optimal fiber length by L^M^_0_, and the muscle pennation angle by α. To increase the reachable space, we adapted the tendon slack length L^T^_s_ of a small number of muscles (bold numbers vs. the original values in brackets).
